# Comparison of psychological characteristics of women with depression and self-harming behavior

**DOI:** 10.1192/j.eurpsy.2021.1827

**Published:** 2021-08-13

**Authors:** T. Medvedeva, S. Enikolopov, O. Vorontsova, O. Kazmina, O. Boyko

**Affiliations:** Clinical Psychology, Federal State Budgetary Scientific institution “Mental health research center”, Moscow, Russian Federation

**Keywords:** Implicit associations, Depression, self-harm, Suicidal risk

## Abstract

**Introduction:**

Existing literature supports the association between depression and self-harm, a prominent risk factor of suicide.

**Objectives:**

Аnalysis of psychological characteristics of women with depression and self-harming behavior and their differences from patients with depression without self-harm.

**Methods:**

The study involved 62 women with depression (age 16–23), 36 with self-harming, 26 did not have episodes of self-harm. Hamilton Scale (HDRS), Wisconsin Card Sorting Test (WCST), Iowa Gambling Task (IGT), SCL-90-R, Rosenberg self-esteem scale, Body Investment Scale (BIS) were used.

**Results:**

Computer test execution time is shorter in the self-harming group, the total time in WCST and IGT tests is significantly shorter (T Test p<0.001), «inhibition» (HDRS) in this group is significantly lower. The self-harmed group demonstrates higher feelings of guilt (2.222±1.141 versus 1.367±1.326 in the non-self-harm group, p=<0.001), suicidal ideation (2.653±1.302 versus 1.100±1.373 p<0.001), psychopathological symptoms in SCL90-R: sensitivity (1.812±0.861 versus 1.185±0.553), hostility (1.388±0.965 versus 0.729±0.700 p=0.004), GSI (1.539±0.705 versus 1.205±0.473 p=0.039), and a special attitude towards body - a decrease of somatic symptoms (HDRS), decreased parameter of “protection” of body and the «attitude to the body» in Body Investment Scale (BIS).

**Conclusions:**

The study revealed psychological characteristics that distinguish a group of depressed women with self-harming: a mismatch of the severity of the components of depressive tirade - motor and ideator inhibition was less pronounced, while the affective component was significantly more pronounced. The body investment is reduced, the need to protect one’s own body is ignored. High level of guilt, and the increased sensitivity characteristic of these patients can be a vulnerability factor.

**Disclosure:**

No significant relationships.

